# Measles and rubella microarray array patches to increase vaccination coverage and achieve measles and rubella elimination in Africa

**DOI:** 10.11604/pamj.supp.2020.35.1.19753

**Published:** 2020-01-03

**Authors:** Lauren Christine Richardson, William John Moss

**Affiliations:** 1 Merrick & Company, Arlington, Virginia, USA; 2 International Vaccine Access Center, Department of International Health, Johns Hopkins Bloomberg School of Public Health, Baltimore, Maryland, USA

**Keywords:** Microarray, measles, vaccine

## Abstract

The African Region is committed to measles elimination by 2020 but coverage with the first dose of measles-containing vaccine was only 70% in 2017. Several obstacles to achieving high coverage with measles and rubella vaccines exist, some of which could be overcome with new vaccine delivery technologies. Microarray array patches (MAPs) are single-dose devices used for transcutaneous administration of molecules, including inactivated or attenuated vaccines, that penetrate the outer stratum corneum of the skin, delivering antigens to the epidermis or dermis. MAPs to deliver measles and rubella vaccines have the potential to be a transformative technology to achieve elimination goals in the African Region. MAPs for measles and rubella vaccination have been shown to be safe, immunogenic and thermostable in preclinical studies but results of clinical studies in humans have not yet been published. This review summarizes the current state of knowledge of measles and rubella MAPs, their potential advantages for immunization programs in the African Region, and some of the challenges that must be overcome before measles and rubella MAPs are available for widespread use.

## Introduction

Global measles vaccination coverage with the first dose of measles-containing vaccine (MCV1) has stagnated at about 85% for the past decade and global goals for reductions in measles incidence and mortality were not met [1]. Although the Region of Americas eliminated measles and rubella (the Americas lost their measles elimination status in 2018), no other World Health Organization (WHO) region has achieved measles elimination despite goals to do so by 2020 or earlier [2]. In 2011, the WHO African Region established a goal to eliminate measles by 2020 [3], but MCV1 coverage in 2017 was only 70% [2], far lower than what is needed for elimination. Numerous obstacles to measles and rubella elimination exist, including conflict, weak immunization systems, insufficient political will and resources and loss of confidence in vaccines leading to decreased demand. Despite regional differences in the underlying causes, the fundamental problem is the same across the globe: failure to achieve high coverage (> 95%) with two doses of measles vaccine. However, the tools to achieve high measles vaccine coverage have not changed much over the past several decades and better vaccine delivery platforms would be beneficial [4]. The only major advance in vaccine delivery since the beginning of the Expanded Program on Immunization in 1974 was the introduction of non-reusable syringes in 2000 [5].

## Methods

We reviewed the published literature on microarray and microneedle patches for vaccine-preventable diseases, with a focus on measles and rubella vaccines. We did not conduct a systematic review of the literature.

## Current status of knowledge

### Microarray patches

**Figure 1:**
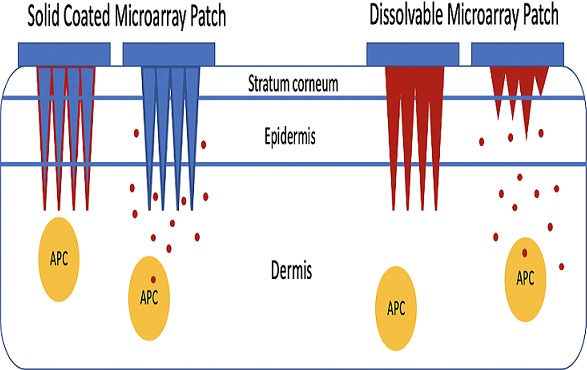
Coated and dissolvable microarray patches for delivery of measles and rubella vaccines; APC: antigen presenting cell

Microarray array patches (MAPs), also known as microneedle patches, are single-dose devices used for transcutaneous administration of molecules, including inactivated or attenuated vaccines, that penetrate the outer stratum corneum of the skin, delivering antigens to the epidermis or dermis [6–8]. MAPs consist of an array of dozens to thousands of micron-sized needles on an adhesive backing (Figure 1). The needles may be solid or hollow, and coated or filled with the vaccine antigens. They can be fabricated from a variety of different materials, including polymers, colloidal silica, ceramics, steel, glass, sugar, hydrogel or alumina. Some array materials, such as polymers, are dissolvable on the skin and polymer blends mixed with vaccine antigens can deliver vaccine antigens to the dermis as they dissolve [9]. MAPs have the potential to be a transformative technology to substantially increase measles and rubella vaccination coverage, achieve regional elimination goals and facilitate global measles and rubella eradication [5, 7, 8]. MAPs offer several potential operational advantages when used for vaccine delivery, including thermostability, improved acceptance, decreased risk of infection, ease of administration, reduced supply chain requirements and medical waste and dose sparing. A critical advantage is the potential improved thermostability of vaccine antigens presented using MAPs because of the use of lyophilized vaccine. Enhanced thermostability could reduce cold chain requirements, minimize loss of vaccine potency and facilitate vaccine delivery in remote rural areas.

Due to the potential for non-painful administration of vaccine antigens (by not stimulating pain receptors deeper within the skin), acceptability may be improved, especially among children. Although data on the acceptability of actual vaccination with MAPs are not yet available, end-user acceptability of a MAP for child immunization was evaluated in a multi-country study of 314 participants in Benin, Nepal and Vietnam using simulated vaccine administration and in-depth interviews [10]. Overall acceptability was 92.7%, but participants recommended that the technology first be introduced at healthcare facilities to establish confidence prior to use for outreach vaccination. In an unpublished study conducted in Ghana, simulated use of a dissolvable MAP by health care workers to vaccinate children and adult women demonstrated acceptability and feasibility, although the time needed to monitor complete vaccine delivery was noted as a potential operational challenge [8]. Another study examined the usability and acceptability of self-administered MAPs [11]. Participants received placebo MAPs three times by self-administration and once by an investigator, in addition to an intramuscular injection of saline to simulate standard vaccination practices. Self-administration was delivered by thumb pressure or a snap-based device. The best usability, as measured by skin staining, was seen with the snap device, with users inserting a median value of 93-96% of microarrays over three repetitions. Most participants (64%) expressed a preference for self-vaccination with MAPs.

Decreased risk of infection could result from the shallow penetration of the microarray needles, as well as the inability of MAPs to be refilled or reused [12]. The delivery technique is easy, requiring minimal training for administration. Importantly, persons not trained as healthcare workers may be able to safely and effectively administer MAPs, facilitating vaccination during mass vaccination campaigns (supplementary immunization activities), outbreaks and in disordered settings such as areas of conflict and other humanitarian emergencies. The logistical requirements for distribution and administration, from supply chain to disposal, may be reduced with MAPs. The volume and weight of shipments for distribution are expected to be lower than most current vaccine products, as no additional materials (e.g. needles, syringes, diluent for reconstitution) are required. Using HERMES modeling software, a simulation study was conducted to assess the impact of MAPs on routine vaccine supply chains in Benin, Bihar and Mozambique [13]. The conclusion was that a MAP would need to have a smaller or equal volume-per-dose than existing vaccine formulations and be able to be stored outside the cold chain for a continuous period of at least two months to provide additional benefits to these supply chains. Because no reconstitution is needed, cold chain requirements are expected to be further lowered and vaccine wastage should be reduced. Hazardous waste also is reduced, as no sharps or biohazardous materials remain after administration. There is potential for the complete absence of biohazardous waste, as dissolvable MAPs are made with water-soluble materials that release vaccine on dissolution [7]. Delivery of vaccine antigens through MAPs may improve immunogenicity, including more robust antibody and cellular response and longer duration of immunity, in part because of the presence of large numbers of antigen-presenting cells in the dermis and epidermis (e.g. dendritic cells) [7]. The potential enhanced immunogenicity of MAP vaccines could result in dose sparing, reducing the cost.

### Studies of vaccine delivery using MAPs

Vaccines delivered through MAPs have undergone preclinical development and testing over the past decade in animal models for several vaccine-preventable diseases, including inactivated poliovirus vaccination in rhesus macaques [14], hepatitis B virus vaccination in mice and rhesus macaques [15], rabies virus vaccination in dogs [16], glycoprotein subunit Ebola virus vaccination in mice [17], formalin-inactivated respiratory syncytial virus vaccination in mice [18] and tetanus toxoid in pregnant mice [19]. Several of these MAP formulations were shown to have increased thermostability compared to currently used vaccines [20]. Although no microarray vaccines have yet been approved by the U.S. Food and Drug Administration (FDA), an FDA-approved solid microarray device can be purchased without vaccine or active ingredient. Much of the published studies using MAPs for vaccine delivery have examined the immunogenicity, safety and thermostability of influenza vaccines [21–24], in part because of the potential market in high-income countries. As an example, dissolving polymer microarray patches were shown in mice to induce antibody and cellular immune responses that provided protection against lethal challenge [22]. Vaccination using dissolvable MAPs resulted in more efficient viral clearance from the lung and enhanced cell-mediated recall responses after viral challenge than standard vaccination, evidence of enhanced immunogenicity when vaccine antigens are delivered into the dermis using MAPs.

Importantly, several published studies investigated influenza vaccination using MAPs in humans. A randomized, partly blinded, placebo-controlled, phase 1, clinical trial enrolled 100 non-pregnant, immunocompetent adults aged 18-49 years [25]. Participants were randomly assigned to four groups and received a single dose of inactivated influenza vaccine by MAP or intramuscular injection, or placebo by MAP, by an unmasked health-care worker. A fourth group received a single dose of inactivated influenza vaccine by MAP self-administered by study participants. The incidence of adverse events was similar across the vaccinated groups and consisted of mild tenderness (60%) and pain (44%) after intramuscular injection, and tenderness (66%), erythema (40%) and pruritus (82%) after vaccination by MAP. Geometric mean antibody titers and the proportion of participants who seroconverted were similar at day 28 between those who received influenza vaccination by MAP, including those who self-administered the patch, compared to intramuscular administration.

A second randomized, partly-blinded, placebo-controlled trial of influenza vaccination in healthy human volunteers was reported using a different MAP (NanopatchTM) [26]. Similar antibody responses were observed between those receiving influenza vaccinations, although sample sizes were small and adverse reactions were reported as mild or moderate. This included pruritis at the site of application, a potential adverse event related to MAPs that is likely due to the vaccine antigen or formulation. The cost-effectiveness of MAPs for influenza vaccination was evaluated in several published studies. A transmission model was coupled to an economic influenza outcomes model to assess the economic value of MAPs for influenza vaccination in the United States [27]. The model suggested that MAPs would be cost-effective or dominant (i.e., less costly and more effective) when administered by health care workers, and also cost-effective when self-administered if they increased compliance sufficiently to overcome any potential reduction in efficacy due to self-administration. Another study examined potential clinical outcomes and direct medical costs of an influenza vaccination program offering a MAP vaccine to children who declined intramuscular vaccine administration in Hong Kong [28]. These studies suggest the potential for MAPs to be cost-effective for influenza vaccination, but the full potential will not be known until MAPs are introduced into practice.

### Measles and rubella vaccination using MAPs

An early study of transcutaneous measles vaccination using a patch in human adult volunteers failed to show induction of neutralizing antibodies, potentially due to the administration method or vaccine dose delivered [29]. However, several published studies have since demonstrated the immunogenicity of measles vaccination using MAPs in animal models. Although different measles MAPs have been developed and tested, the most promising consists of 100 microscopic water-soluble polymer cones, each the width of a human hair, that contain currently available, lyophilized, attenuated measles vaccine and that dissolve into the skin within several minutes of application. Following studies showing immunogenicity and safety in cotton rats [30], measles vaccine delivered via polymeric microarrays was shown to be immunogenic in rhesus macaques [31]. The dissolvable MAPs included the encapsulated, standard dose of the Edmonston-Zagreb vaccine strain (1000 TCID50) applied for 10 minutes, resulting in production of neutralizing antibody titers equivalent to those generated following standard subcutaneous vaccine administration with no adverse events except mild skin erythema. Importantly, the measles MAP demonstrated thermostability at 4-8oC for four months without unacceptable loss of potency, evidence of enhanced thermostability.

Because of the programmatic importance of concurrent administration of measles and rubella vaccines, a monovalent measles vaccine delivered by a MAP is unlikely to be widely used. Importantly, immunogenicity and safety were also demonstrated using the same MAP to deliver combined measles (Edmonston-Zagreb strain) and rubella (RA-27 strain) virus antigens in infant rhesus macaques [32]. Protective neutralizing antibody titers were detected in all infant macaques following vaccination with the measles-rubella MAP but in only 75% of infant macaques following subcutaneous vaccination, again evidence of enhanced immunogenicity. These antibody titers resulted in protection against wild-type measles virus challenge. Rubella neutralizing antibody titers were >10 IU/mL, the minimum protective level, for both groups of infant macaques. However, protective titers against measles were not achieved following either MAP or subcutaneous vaccine administration in macaques pretreated with immunoglobulin, simulating maternal antibodies, suggesting MAPs are not able to overcome the inhibitory effect of pre-existing, maternal neutralizing antibodies. These MAPs dissolved completely upon skin penetration and were thermostable for one month at 40oC, exceeding World Health Organization stability requirements. No adverse effects were noted.

The potential cost-effectiveness of a measles MAP was assessed using a spreadsheet model to compare the vaccination costs of MAPs with vaccine administration through needles and syringes, assuming MAPs would be more thermostable with less requirements for a cold chain [33]. Measles MAPs were estimated to cost US$0.95 per dose compared with US$1.65 for standard measles vaccine administered subcutaneously. Assuming these costs and 95% measles vaccine coverage with the first measles vaccine dose, MAPs were estimated to cost US $1.66 per measles case averted compared to US $2.64 per case averted with subcutaneous vaccination. The cost-effectiveness of MAPs will ultimately depend on cost, acceptability and effectiveness when implemented in immunization programs.

### MAPs for measles vaccination in Africa

The use of MAPs for administration of measles and rubella vaccines in Africa could be particularly advantageous and potentially transformative [7, 8]. First, increased thermostability of a measles-rubella MAP could reduce cold chain requirements and facilitate transportation of the vaccine to remote areas in rural sub-Saharan Africa. Second, a measles-rubella MAP could be administered by minimally trained personnel (or even self-administered), making house-to-house measles and rubella vaccination campaigns possible using community health workers or other trained community members. Third, a measles-rubella MAP would not require reconstitution, obviating the need for needles and syringes and eliminating human error in reconstitution of the lyophilized vaccine (e.g. use of the incorrect diluent or volume, or bacterial contaminated diluent). Fourth, a measles-rubella MAP would overcome hesitation in opening a multidose vaccine for one or a few children, minimizing missed opportunities for vaccination and vaccine wastage. Fifth, a measles-rubella MAP would eliminate needle stick injuries and reuse of needles and syringes. Sixth, a dissolvable measles-rubella MAP would minimize biohazardous medical waste. Seventh, supply chain requirements could be reduced, due to lower cargo weight (no glass vials), lower cold chain volumes, and no need for consumable compatibility (e.g. needles and syringes that are compatible). Lastly, a painless measles-rubella MAP could improve acceptability in some communities.

### The future of MAPs

Despite the potential advantages of MAPs for delivery of measles and rubella vaccines in sub-Saharan Africa, several challenges must be overcome before MAPs could be available for widespread use. The most significant obstacle relates to the value proposition of MAPs for measles and rubella vaccine delivery given the costs of development, manufacturing and use in immunization programs [8]. Thus, the product attributes of MAPs will need to confer substantial advantages to justify these investments, with a clear market demand to demonstrate the return on investment. MAPs can currently be produced on a small scale to support evaluation in early phase clinical trials but large scale production under Good Manufacturing Production (GMP) conditions will require a significant investment and several years from planning to production [8]. A key issue is whether MAPs need to be manufactured aseptically, as they are ultimately applied under non-sterile conditions, or whether demonstrated safety with low bioburden material would be acceptable [8]. Whether the investment in large-scale production facilities occurs concurrently with clinical trials of safety and efficacy, or is delayed until after phase 3 clinical trials are completed, will strongly determine the timeline as to when MAPs could be available for use in Africa.

There are also regulatory pathways that must be completed based on safety and efficacy data. A measles and rubella MAP would likely be considered a new product by regulatory agencies, despite the fact that currently used measles and rubella vaccine strains would comprise the antigenic components [8]. The measles and rubella vaccine formulations may need to be modified to optimize delivery through coated or dissolvable MAPs [8]. Because measles and rubella vaccines have generally accepted immunologic correlates of protection, demonstration of immunologic non-inferiority of a MAP (i.e. similar antibody titers within a pre-defined margin) compared to standard subcutaneous administration of measles and rubella vaccines may be sufficient. Ultimately, a MAP to deliver measles and rubella vaccines in sub-Saharan Africa will require WHO pre-qualification. The European Medicines Agency’s Article 58, a regulatory pathway for innovative vaccines for diseases of public health importance, could facilitate prequalification by the WHO and registration in African countries [8]. Nevertheless, the financial, manufacturing, and regulatory hurdles mean that the availability of MAPs for measles and rubella vaccination is at least five years and probably longer from realization.

Importantly, to shorten this process as much as possible, the minimum and preferred attributes for a MAP to deliver measles and rubella vaccines are being developed by the WHO and an expert working group, leading to a target product profile. Efforts such as the Vaccination Innovation Prioritization Strategy, a partnership comprised of WHO, Gavi, the Bill & Melinda Gates Foundation, PATH and UNICEF, and PATH’s Center of Excellence for Microarray Patch Technology, are critical efforts to accelerate the development of MAPs and provide guidance on research, regulatory pathways and manufacturing conditions. Hopefully, these efforts will expedite the development, testing, manufacturing, and implementation of MAPs for measles and rubella vaccination in immunization programs.

## Conclusion

MAPs to deliver measles and rubella vaccines could play a critical role in achieving elimination goals in the African Region. Key stakeholders, including policy makers, ministers of health and finance, vaccine advocates, and immunization program managers, need to be aware of this potentially transformative technology and have a voice in moving the product development pipeline forward.

### What is known about this topic

- Global MCV1 coverage has stagnated at 85% and is only 70% in the African Region;
- Currently used measles and rubella vaccines are safe, effective and low cost but several obstacles exist to achieving high vaccination coverage;
- These obstacles include the need to maintain a cold chain and use skilled health care workers, and the potential for missed opportunities and vaccine wastage.

### What this study adds

- Microarray patches to deliver measles and rubella vaccines have the potential to be a transformative technology to achieve elimination goals in the African Region;
- Microarray patches for measles and rubella vaccination have been shown to be safe, immunogenic and thermostable in preclinical studies;
- Several obstacles must be overcome before MAPs are available for measles and rubella vaccination, including investment in large-scale production facilities and obtaining WHO pre-qualification.

## Competing interests

The authors declare no competing interests.

## References

[1] Moss WJ (2017). Measles. Lancet.

[2] Dabbagh A, Laws RL, Steulet C, Dumolard L, Mulders MN, Kretsinger K (2018). Progress toward regional measles elimination - Worldwide, 2000-2017. MMWR.

[3] Masresha BG, Dixon MG, Kriss JL, Katsande R, Shibeshi ME, Luce R (2017). Progress toward measles elimination - African Region, 2013-2016. MMWR.

[4] Giersing BK, Kahn AL, Jarrahian C, Mvundura M, Rodriguez C, Okayasu H (2017). Challenges of vaccine presentation and delivery: How can we design vaccines to have optimal programmatic impact?. Vaccine.

[5] Durrheim DN, Goodson JL (2017). Time for an immunisation paradigm shift. Transactions of the Royal Society of Tropical Medicine and Hygiene.

[6] Zheng Z, Diaz-Arevaloc D, Guana H, Zenga M (2018). Noninvasive vaccination against infectious diseases. Human Vaccines & Immunotherapeutics.

[7] Arya J, Prausnitz MR (2016). Microneedle patches for vaccination in developing countries. Journal of Controlled Release.

[8] Peyraud N, Zehrung D, Jarrahian C, Frivold C, Orubu T, Giersing B (2019). Potential use of microarray patches for vaccine delivery in low- and middle- income countries. Vaccine.

[9] Li J, Zeng M, Shan H, Tong C (2017). Microneedle patches as drug and vaccine delivery platform. Current Medicinal Chemistry.

[10] Guillermet E, Alfa DA, Phuong Mai LT, Subedi M, Demolis R, Giersing B (2019). End-user acceptability study of the nanopatch; a microarray patch (MAP) for child immunization in low and middle-income countries. Vaccine.

[11] Norman JJ, Arya JM, McClain MA, Frew PM, Meltzer MI, Prausnitz MR (2014). Microneedle patches: usability and acceptability for self-vaccination against influenza. Vaccine.

[12] Donnelly RF, Singh TR, Tunney MM, Morrow DI, McCarron PA, O’Mahony C (2009). Microneedle arrays allow lower microbial penetration than hypodermic needles in vitro. Pharmaceutical Research.

[13] Wedlock PT, Mitgang EA, Elsheikh F, Leonard J, Bakal J, Welling J (2019). The potential effects of introducing microneedle patch vaccines into routine vaccine supply chains. Vaccine.

[14] Edens C, Dybdahl-Sissoko NC, Weldon WC, Oberste MS, Prausnitz MR (2015). Inactivated polio vaccination using a microneedle patch is immunogenic in the rhesus macaque. Vaccine.

[15] Perez Cuevas MB, Kodani M, Choi Y, Joyce J, O’Connor SM, Kamili S (2018). Hepatitis B vaccination using a dissolvable microneedle patch is immunogenic in mice and rhesus macaques. Bioengineering & Translational Medicine.

[16] Arya JM, Dewitt K, Scott-Garrard M, Chiang YW, Prausnitz MR (2016). Rabies vaccination in dogs using a dissolving microneedle patch. Journal of Controlled Release.

[17] Liu Y, Ye L, Lin F, Gomaa Y, Flyer D, Carrion R (2018). Intradermal immunization by Ebola virus GP subunit vaccines using microneedle patches protects mice against lethal EBOV challenge. Scientific Reports.

[18] Park S, Lee Y, Kwon YM, Lee YT, Kim KH, Ko EJ (2018). Vaccination by microneedle patch with inactivated respiratory syncytial virus and monophosphoryl lipid A enhances the protective efficacy and diminishes inflammatory disease after challenge. PloS One.

[19] Esser ES, Romanyuk A, Vassilieva EV, Jacob J, Prausnitz MR, Compans RW (2016). Tetanus vaccination with a dissolving microneedle patch confers protective immune responses in pregnancy. Journal of Controlled Release.

[20] Kolluru C, Gomaa Y, Prausnitz MR (2019). Development of a thermostable microneedle patch for polio vaccination. Drug Delivery and Translational Research.

[21] Kim YC, Quan FS, Yoo DG, Compans RW, Kang SM, Prausnitz MR (2009). Improved influenza vaccination in the skin using vaccine coated microneedles. Vaccine.

[22] Sullivan SP, Koutsonanos DG, Del Pilar Martin M, Lee JW, Zarnitsyn V, Choi SO (2010). Dissolving polymer microneedle patches for influenza vaccination. Nature Medicine.

[23] Kommareddy S, Baudner BC, Bonificio A, Gallorini S, Palladino G, Determan AS (2013). Influenza subunit vaccine coated microneedle patches elicit comparable immune responses to intramuscular injection in guinea pigs. Vaccine.

[24] Jacoby E, Jarrahian C, Hull HF, Zehrung D (2015). Opportunities and challenges in delivering influenza vaccine by microneedle patch. Vaccine.

[25] Rouphael NG, Paine M, Mosley R, Henry S, McAllister DV, Kalluri H (2017). The safety, immunogenicity, and acceptability of inactivated influenza vaccine delivered by microneedle patch (TIV-MNP 2015): a randomised, partly blinded, placebo-controlled, phase 1 trial. Lancet.

[26] Fernando GJP, Hickling J, Jayashi Flores CM, Griffin P, Anderson CD, Skinner SR (2018). Safety, tolerability, acceptability and immunogenicity of an influenza vaccine delivered to human skin by a novel high-density microprojection array patch (Nanopatch). Vaccine.

[27] Lee BY, Bartsch SM, Mvundura M, Jarrahian C, Zapf KM, Marinan K (2015). An economic model assessing the value of microneedle patch delivery of the seasonal influenza vaccine. Vaccine.

[28] Wong C, Jiang M, You JH (2016). Potential cost-effectiveness of an influenza vaccination program offering microneedle patch for vaccine delivery in children. PloS One.

[29] Etchart N, Hennino A, Friede M, Dahel K, Dupouy M, Goujon-Henry C (2007). Safety and efficacy of transcutaneous vaccination using a patch with the live-attenuated measles vaccine in humans. Vaccine.

[30] Edens C, Collins ML, Ayers J, Rota PA, Prausnitz MR (2013). Measles vaccination using a microneedle patch. Vaccine.

[31] Edens C, Collins ML, Goodson JL, Rota PA, Prausnitz MR (2015). A microneedle patch containing measles vaccine is immunogenic in non-human primates. Vaccine.

[32] Joyce JC, Carroll TD, Collins ML, Chen MH, Fritts L, Dutra JC (2018). A Microneedle Patch for Measles and Rubella Vaccination Is Immunogenic and Protective in Infant Rhesus Macaques. The Journal of Infectious Diseases.

[33] Adhikari BB, Goodson JL, Chu SY, Rota PA, Meltzer MI (2016). Assessing the potential cost-effectiveness of microneedle patches in childhood measles vaccination programs: the case for further research and development. Drugs in R & D.

